# Historical Analysis Exposes Catastrophic Seagrass Loss for the United Kingdom

**DOI:** 10.3389/fpls.2021.629962

**Published:** 2021-03-04

**Authors:** Alix E. Green, Richard K. F. Unsworth, Michael A. Chadwick, Peter J. S. Jones

**Affiliations:** ^1^Department of Geography, University College London (UCL), London, United Kingdom; ^2^Seagrass Ecosystem Research Group, Swansea University, Swansea, United Kingdom; ^3^Project Seagrass, Sustainable Places Research Institute, Cardiff, United Kingdom; ^4^Department of Geography, King’s College London, London, United Kingdom

**Keywords:** blue carbon, ecosystem change, habitat loss, intertidal, historic change, marine, shifting baseline syndrome, Zostera spp.

## Abstract

The spatial extent of seagrass is poorly mapped, and knowledge of historical loss is limited. Here, we collated empirical and qualitative data using systematic review methods to provide unique analysis on seagrass occurrence and loss in the United Kingdom. We document 8,493 ha of recently mapped seagrass in the United Kingdom since 1998. This equates to an estimated 0.9 Mt of carbon, which, in the current carbon market represents about £22 million. Using simple models to estimate seagrass declines triangulated against habitat suitability models, we provide evidence of catastrophic seagrass loss; at least 44% of United Kingdom’s seagrasses have been lost since 1936, 39% since the 1980’s. However, losses over longer time spans may be as high as 92%. Based on these estimates, historical seagrass meadows could have stored 11.5 Mt of carbon and supported approximately 400 million fish. Our results demonstrate the vast scale of losses and highlight the opportunities to restore seagrass to support a range of ecosystems services.

## Introduction

Increased interest in the Blue Carbon capacity of seagrass means knowledge of the location, extent, and condition of seagrasses has become increasingly important (Fourqurean et al., 2012; Greiner et al., 2013; Röhr et al., 2018; Green et al., 2018). Seagrasses are highly productive, and represent one of the largest global carbon sinks despite occupying only 0.1% of the ocean floor (Hemminga and Duarte, 2000; Orth et al., 2006; Fourqurean et al., 2012; Duarte et al., 2013). An estimated 19.9 Pt carbon is stored in the top 1 m of seagrass worldwide, the equivalent to the CO_2_ emissions from fossil fuel and cement production in 2014 (Kerr, 2017). Seagrasses also support biodiversity, as well as contributing to the productivity of 20% of the world’s biggest fisheries (Unsworth et al., 2019), supporting coastal livelihoods, increasing shoreline stability, cycling nutrients, and making our coastlines make more affable places to live (Cullen-Unsworth et al., 2014; Nordlund et al., 2016).

Under the Paris agreement countries pledged to outline National Determined Contributions (NDC’s) to reduce their emissions (Martin et al., 2016), and nature-based solutions are increasingly being adopted within these strategies. To date, six countries name seagrass directly in their NDCs (Martin et al., 2016). Although these inclusions are encouraging, to the best of our knowledge, there has been no attempt by any country to document the total areal extent and historic loss of seagrass in their coastal waters. One of the main global challenges of seagrass conservation is that the status of many seagrass meadows is unknown (Unsworth et al., 2019). Knowing how much seagrass a country has is clearly an important step to knowing how to protect it; but knowing where seagrass was, or where seagrass could thrive, gives countries an opportunity to re-plant and restore seagrass in favourable areas (Cunha et al., 2012; Greiner et al., 2013; Paulo et al., 2019).

It is increasingly accepted that restoration of natural habitats must play a crucial role in global efforts to mitigate climate change (European Commission, 2009). That seagrasses can absorb more carbon up to 40 faster than terrestrial forests (Mcleod et al., 2011) should make them a significant component of these attempts. Global loss of seagrasses since the 1980’s is thought to be at least 29% (Waycott et al., 2009; Short et al., 2010), and seagrasses continues to be lost at a rate of 1.4% a year (Short et al., 2010). These losses must be stemmed if seagrasses are to play a role in climate mitigation and understanding where losses have occurred is an important first step towards appropriate conservation planning (Cullen-Unsworth and Unsworth, 2016).

Seagrasses are highly sensitive to degrade water quality and conditions which impose light limitations to photosynthesis (Orth et al., 2006). Coastal development and nutrient enrichment have historically been responsible for worldwide declines, which threaten the substantial ecological services seagrass meadows provide (Fraser and Kendrick, 2017). Global seagrass declines only account for mapped populations, and in many countries, data on extent are limited. Even in developed countries, such as those of the United Kingdom, spatial data on seagrass extent are largely incomplete. Given the paucity of seagrass mapping to date, the baseline from which global seagrass declines are calculated are almost certainly significant underestimations. The most up-to-date estimate of seagrass coverage indicate that a minimum of 325,178 km^2^, occurs globally, but these values include data from the United Kingdom that is largely out of date (Short, 2018; McKenzie et al., 2020). Recent efforts have been made to demonstrate the substantial services afforded by United Kingdom seagrass habitats through sediment stabilisation (Wilkie, 2011), fisheries support (Bertelli and Unsworth, 2014), and carbon sequestration (Green et al., 2018). Understanding the significance of these services is challenging without robust estimates of the current and historic areal extent of United Kingdom seagrass meadows.

As with global seagrass observations, monitoring and mapping of United Kingdom seagrasses occurs with limited consistency (McKenzie et al., 2020). Where studies have occurred, the resulting data are largely in the grey literature or held disparately by local councils, national and devolved governments, and non-government organisations (NGO’s). This has resulted in a lack of current and robust estimates on spatial coverage of seagrasses. The needs for these estimates are multiple but recent studies highlight the poor status of seagrasses in the United Kingdom (Jones and Unsworth, 2016).

Once considered a significant component of the natural heritage of United Kingdom waters (Davidson and Hughes, 1998), seagrass is now accepted to be nationally scarce and sparsely distributed (Hiscock et al., 2005; Jones and Unsworth, 2016). Conceptions of environmental degradation tend to shift depending on our temporal reference point. In the United Kingdom, this “shifting baseline syndrome” (SBS; Pauly, 1995) occurs when the earliest known data of areal extent are assumed as an unaffected baseline condition. This is further exacerbated by data being supported by qualitative accounts that refer to healthier conditions within a scientist’s lifetime (Butcher, 1941; Pauly, 1995). With each generation, the concept of a healthy ecosystem shifts, depending on their perceived baseline.

The earliest attempts to document seagrass extent already pointed to declines and the need for more data (Butcher, 1934, 1941). It is likely that Butcher’s 1930’s reports were already subject to SBS. Two periods of decline are emphasised throughout the literature: one immediately after WWI, and another during the northern Atlantic outbreak of wasting disease in the early 1930’s (Butcher, 1934; Cottam, 1934). The wasting disease “epidemic” has been perpetually attributed as the main cause of declines (Den Hartog, 1983; Garrard and Beaumont, 2014) without consideration for the pervasive environmental degradation that occurred in the centuries before. Regardless of the cause of these declines, more efforts are needed to evaluate the status and trends of these valuable marine habitats. To fully appreciate the extent of declines, we must find a way to look beyond these earliest evaluations, which are almost certainly underplayed due to SBS. The objectives of this study were accordingly to estimate for the United Kingdom: (1) the current areal extent of seagrass; and (2) the recent (since 1998) and historic (since before 1998) percentage loss of seagrass. The paper places our results in the context of conservation and provision of ecosystem services.

## Materials and Methods

The United Kingdom contains two species of seagrass; *Zostera marina* and *Zostera noltii*. The former is predominately sublittoral, and the latter occurs intertidally (Wilkinson and Wood, 2003). Both species are protected under the EU Habitats Directive (92/43/EEC) as features included in annex 1 (habitats), are indicators of Good Ecological Status under the EU Water Framework Directive (WFD; Foden and Brazier, 2007), and gain protection from a number of other EU Directives due to their need for good water quality [i.e., EU Nitrates Directive (91/676/EEC), Urban Wastewater Treatment Directive (91/271/EEC)], and their importance as habitat for wildfowl [Birds Directive (79/409/EEC); Jackson et al., 2016]. In addition, seagrass habitats across a range of United Kingdom waters are offered protection associated with amend and devolved legislation stemming from the Wildlife and Countryside Act 1981.

For the purpose of this work, we have categorised any data collected since 1998 as “contemporary” (similar to current conditions), and any data older as “historical” (not reflecting current conditions), since we cannot reliably confirm the presence of something that has not been mapped for over 20 years. To fulfill the first objective, multiple datasets were collated with other isolated data, to determine the current mapped areal extent of seagrasses in the United Kingdom. Due to the paucity of available data, we have used three methods to assess seagrass loss with high, medium, and low certainty. High certainty loss estimates were generated collating data older than 1998 and cross-checking them against contemporary data to confirm loss of areal extent. Medium certainty loss includes data on sites where no contemporary data are available, i.e., sites that have not been revisited since 1998. All these methods were supplemented by a systematic review to provide qualitative and quantitative data on seagrass loss. Low certainty loss estimates, not subject to SBS and data limitations, were derived using best available data on historic seagrass extent and additional data regarding sub- and intertidal, mud- and sandflat area of England, Scotland, and Wales (mainland Britain) to estimate maximum seagrass extent and percentage loss in mainland Britain. These estimates excluded Ireland and Northern Ireland because accurate data on mud- and sandflat area were not available.

### Contemporary and Historical Areal Estimates of Seagrass Habitats

Two datasets were identified as containing records of *Zostera* from multiple sites. The OSPAR Commission (2017) dataset represents the current known areal extent of seagrasses in the United Kingdom and includes records on *Zostera* between 1986 and 2015. Under the WFD, a range of government agencies (e.g., English & Northern Ireland Environment Agency, Scottish Environment Protection Agency, and Natural Resources Wales) are required to assess the condition of seagrass to help determine the biological condition of United Kingdom water bodies (Foden and Brazier, 2007). The outcome of this is another dataset that includes areal extent of *Zostera* meadows monitored under the WFD between 2007 and 2017. These data were analysed by region and date on QGIS (version 3.2.1) and were shown to contain substantial gaps. To supplement these, we contacted stakeholders from a multitude of organisations targeting local councils, national and devolved governments, government advisory organisations, fisheries authorities, private environmental consultants, and scientists who work on seagrasses in the United Kingdom. From these searches, 14 additional contributors supplemented the OSPAR and WFD datasets, the collective of which makes up all the known available data, based on the searches we undertook (see Supporting information). Since species identification was not provided across all data sets, they have not been included here. However, as an intertidal species, there are far less technological constraints associated with surveying *Z. noltii*. Because of this we expect it to be in the majority, and further expect many *Z. marina* meadows to have gone unreported. Because of these inconsistencies, we have decided not to discriminate between species, since greater abundance of one species is likely due to mapping inconsistencies rather than variances in the conditions that allow one species to proliferate over another. It should also be noted that *Zostera angustifolia* was once considered its own species, although is now recognised as a phenotype of *Z. marina* (Becheler et al., 2010). As we do not distinguish between species, *Z. angustifolia* is treated in the same way as *Z. marina* and *Z. noltii*.

Data were provided in the form of individual observations (point data) and area estimates (polygon data). Polygon data were used to provide the area estimates contained herein. Spatial assessments were made using QGIS (version 3.2.1) and all data were analyzed for duplicates or overlaps. Where they occurred the most recent data was used, unless differences between years occurred that represented data collection restraints rather than area changes. For example, when data were present in the same location from 2016 to 2018 but the 2018 data held substantially reduced area, it was assumed that this was not representative of habitat degradation but restrictions to accessing the full extent of the meadow. This was supported by several data fluctuating between 3 years – e.g., 2014 and 2016 showed the same area cover, but 2015 showed far reduced cover. The area of each polygon was calculated in m^2^ using a cylindrical WGS-84 projection and converted to hectares (ha) for reporting purposes.

The contemporary data represent the minimum area of seagrasses in the United Kingdom since some meadows have certainly gone unreported. OSPAR data were used to provide high and medium certainty estimates of historic mapped areal extent. The maximum seagrass extent for each record within the dataset was checked against contemporary records and where contemporary records were found the difference between largest (oldest) and current meadow size was used to provide high certainty loss estimates. Where no contemporary record of the meadow was found, these were considered as spatial loss and included in medium certainty loss estimates. We acknowledge our approach, and the subsequent estimates of changes in coverage through time, is constrained by sampling efforts and data reporting of past research efforts, but this represents the best use of the best available data.

### Systematic Review of Qualitative and Quantitative Data on Seagrass Declines

Systematic reviews are used to encapsulate a broad range of literature on a discrete subject by aggregating large data and rigorously extracting relevant information (Minx et al., 2017). We followed the distinct protocols required to achieve a systematic review by: (1) defining the discrete subject parameters and the timeframe of interest; (2) creating a search term to encapsulate all data that might be relevant to the subject; (3) inputting this into Web of Science (Thompson Reuters) to extract a literature database; (4) justifying and making a transparent selection of the literature; and (5) providing a synthesis of the relevant literature. Full details of these methods are provided in the supporting information (SI).

Web of Science includes published, peer-reviewed articles as far back as 1990. Because of the distinct lack of published data on seagrass area cover in the United Kingdom, and a need to capture data as far back in time as possible, it was necessary to broaden the search to include published, unpublished, and grey literature. These data were collected by extensive internet searches, through contacting stakeholders from government, private organisations, and NGO’s, and from scientists and the public who work in seagrass science, conservation, and management throughout the United Kingdom. Papers were qualified and data extracted based on the same criteria as the systematic search (SI). Both search unearthed 179 papers that were considered relevant to this work.

### Modelling the of Seagrass Throughout England, Scotland, and Wales

Because of the scarcity of historic empirical data, and the observation that many of the early qualitative reports are almost certainly subject to SBS, we used available data to model the maximum historic extent and low certainty loss estimates of seagrasses in mainland Britain.

**Figure 1:**
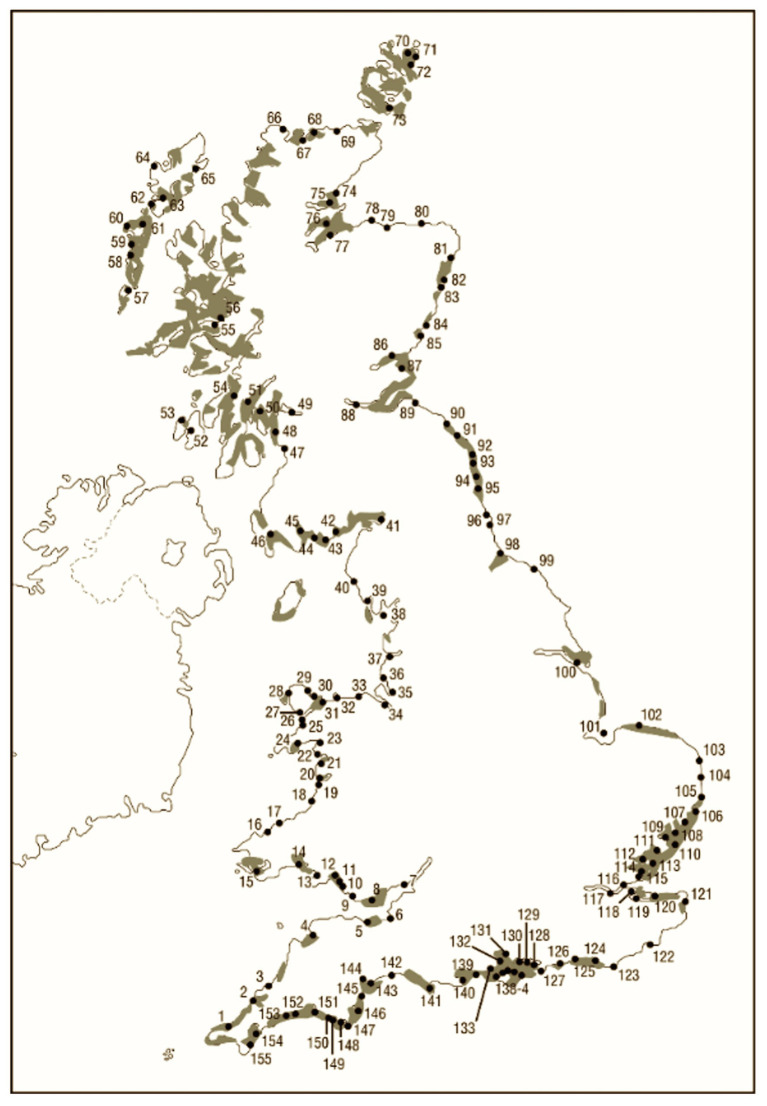
Butcher’s 1930s estimate of seagrass area cover (shaded; Butcher, 1934) and numbers referring to estuaries identified by Nature Conservancy Council (Davidson et al., 1991; image created by UCL drawing office).

In 1991, the Nature Conservancy Council (NCC) undertook a report on the 155 estuaries that exist in mainland Britain (Davidson et al., 1991). In 1932, Butcher reported on the distribution of *Zostera* in the United Kingdom, including a spatial distribution map of mainland Britain that corresponds very closely with the estuaries map presented in the NCC report (Figure 1; Butcher, 1934). Further, qualitative data suggest that before WWI seagrass would once have been found across a large proportion of subtidal mud- and sandflats and on the lower ranges of most intertidal flats throughout the United Kingdom, especially prevalent in estuaries (Cotton, 1933; Butcher, 1934, 1941; Cottam, 1934). Sub and intertidal mud- and sand-flats, in particular, estuarine ones are, therefore, a good proxy for modelling historic seagrass distribution.

We identified locations with best available data on seagrass area cover from either historic or contemporary estimates, where contemporary estimates represent meadows that are in reasonably good condition, and where total mud- /sandflat area for each site was available. This restricted the inclusion of sites to those designated as Special Protected Areas (SPA’s) and Special Areas of Conservation (SAC’s; JNCC, 2005, 2015, 2018) since such sites have been accurately mapped by the Joint Nature Conservation Committee (JNCC; Table 1).

**Table 1:** Seagrass meadow area data used to calculate historic seagrass loss in the United Kingdom.

Site name	Site location	Current extent ha	Historic extent ha	m/s-flat area ha	Seagrass area/ha m/s-flat	Reference for m/s-flat area
Spurn bight	Humber estuary	0.59	550	4,842	0.11	JNCC, 2018
Lindisfarne	NE England	679	1,046	1,571	0.67	JNCC, 2015
Foulness/maplin sands	Thames estuary	40.	320	8,746	0.04	JNCC, 2005
Fal and helford	Cornwall	104	208	645	0.32	JNCC, 2018
River stour and orwell	Thames estuary	1	380	2,620	0.15	JNCC, 2018
Exe estuary	Cornwall	146	**N.D.**	900	0.16	JNCC, 2018
Dornoch firth	East Scotland	117	2,546	6,787	0.38	JNCC, 2018
Cromarty firth	East Scotland	1,200	3,241	3,766	0.86	JNCC, 2018
Moray firth	East Scotland	**N.D.**	1,098	2,339	0.47	JNCC, 2018
Plymouth sound	Devon	92	**N.D.**	2,555	0.04	JNCC, 2018

Current and historic extent (where available) and mud-/sandflat (m/s-flat) area were used to determine average seagrass area per hectare (ha) of mud-/sandflat. N.D. = no available data

**Figure 2:**
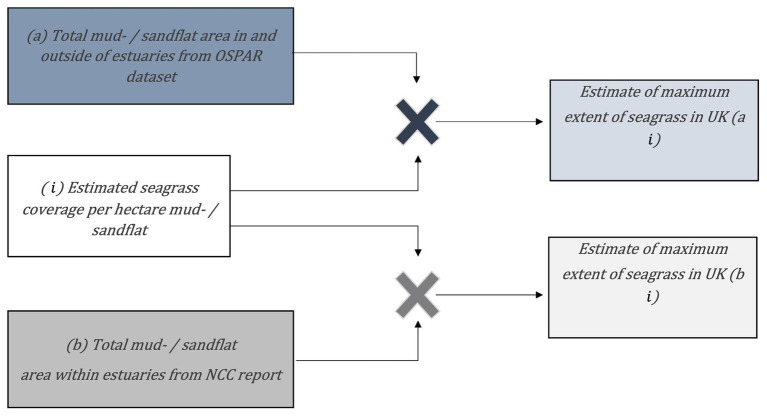
Calculations used to estimate maximum areal extent of seagrass in mainland Britain:a×i=ai;b×i=bi. i is the average seagrass area from 11 sites with good historic or contemporary estimates divided by known mud- and sand flat area.

The chosen sites represent typical environmental variation for United Kingdom seagrasses including intertidal and subtidal habitats within estuaries, rivers, lochs, and spits. Seagrass area coverage per hectare of mud- / sandflat was calculated for each site by dividing total seagrass area by total mud- /sandflat area (Table 1; Figure 2i). Seagrass coverage was estimated using bootstrapping techniques (Manly, 2006). Our 10 sites were randomly selected with replacement 1,000 times and multiplied by total mud-/sandflat area estimates from (a) the OSPAR dataset (2017), which includes data in and outside of estuaries, and (b) the NCC report (Davidson et al., 1991), which includes data from estuaries only (Figures 2a,b). The average of these 1,000 estimates were used to estimate maximum extent of seagrasses around mainland Britain (Figures 2ai,bi). We used this simple bootstrapping procedure (Manly, 2006), rather than more typical parametric statistical methods, due to the paucity of available data. The data provide low certainty maximum seagrass extent around mainland Britain. In addition to the simple modelling approach, datasets were also obtained from previous studies using habitat suitability modelling to estimate potential seagrass distribution (Brown, 2015; Defra, 2020).

## Results

### Contemporary and Pre-1998 Areal Estimates of Seagrass Habitats

The total mapped areal extent of contemporary seagrass records (post-1997) from the OSPAR dataset, the WFD dataset, and all other contributors includes 47 surveys spanning 20 years, 79% of which are from the last 10 years (see Supporting Information). In total, the data confirm the presence of 8,493 ha of seagrasses in the United Kingdom (Table 2). Occurrence of seagrasses is not uniform. Half of all mapped seagrass occurs in the Scottish Highlands (20%), Devon (16.2%), and Northern Ireland (14.3%; Table 2). Seagrass occurrence ranges from patches less than 1m^2^ to meadows up to 1,200 ha (i.e., Cromarty Firth, East Scotland). The average size of seagrass record is 2.64 ± 32.22 ha.

**Table 2:** Distribution of contemporary mapped seagrass area from the OSPAR and Water Framework Directive datasets, and other collected data sources since 1998.

Location	Area ha	% of total
Scottish highlands	2,056	24.21
Devon	1,392	16.39
Northern Ireland	1,810	14.44
Hampshire and Isle of wight	714	8.41
Northumbria	680	8.01
South Wales	460	5.42
Dorset	372	4.38
Scilly Isles	196	2.31
North Wales	172	2.03
Suffolk, Essex, and Kent	170	2.00
Cornwall	166	1.95
East Scotland	108	1.27
West Wales	90	1.06
Cumbria	65	0.77
Norfolk	42	0.49
**Total**	**8,493**	

Data present total known areal extent of seagrass in the United Kingdom by region, including relative contribution to the total mapped area.

The OSPAR dataset, which represents the currently used known areal extent of seagrasses in the United Kingdom, includes 13,753 ha of seagrass. Of this, 8,835 ha (64.2%) was recorded pre-1998 and 4,919 ha (35.76%) was contemporary (post-1997). Within the OSPAR dataset, there is an inverse relationship between average meadow size relative to age of record, i.e., the average area of historic seagrass meadows is 71 ± 218 ha whilst the average area of contemporary seagrass meadows is 2 ± 238 ha. The average for all the meadows included in the OSPAR dataset between 1986 and 2015 is 4 ± 50 ha. GIS point data exist beyond the extent of these mapped areas but the extent of any seagrasses associated to such an observation is unknown and therefore not included within this analysis.

The total mapped historic extent of seagrasses in the United Kingdom is 16,524 ha. The total documented loss of seagrasses since 1936 is 6,697 ha. A further 1,364 ha of seagrass habitat has not been revisited since 1998 (Table 3), a disproportionate amount of which is from Scotland (Table 4). With high certainty the UK has, therefore, lost 44% of its seagrass since 1936, 34% since the 1980s. With medium certainty, including historic data with no recent observations, 50% has been lost since 1936, and 42% since the 1980s.

**Table 3:** High certainty seagrass loss (by area and percent reduction) in regions where good historic data are available based on the systematic review.

	Max extent (pre-1998)	Contemporary area	High certainty loss since 1936	
	*ha*	*ha*	*ha*	*%*
UK	16,524	8,335	6,826	41
Cornwall	271	166	167	62
Essex	450	170	280	62
Northumbria	1,595	679	916	57
NW England	224	65	159	71
Scilly Isles	325	196	129	40
Scotland	8,312	2,164	4,790	58
Suffolk	380	1	379	100

**Table 4:** Estimated seagrass loss from high (known) and medium (unmapped) estimates across all regions and Scotland, calculated by analysing data older than 1998 from the OSPAR dataset.

	High certainty, known seagrass loss	Total unmapped seagrass	Medium certainty seagrass loss
All regions	6,697	1,364	8,061
Scotland	4,790	1,358	6,148

Known loss is from sites which have been revisited and data captured since 1998, unmapped is from sites that have not been revisited.

### Systematic Review of Qualitative and Quantitative Data on Seagrass Declines

**Figure 3:**
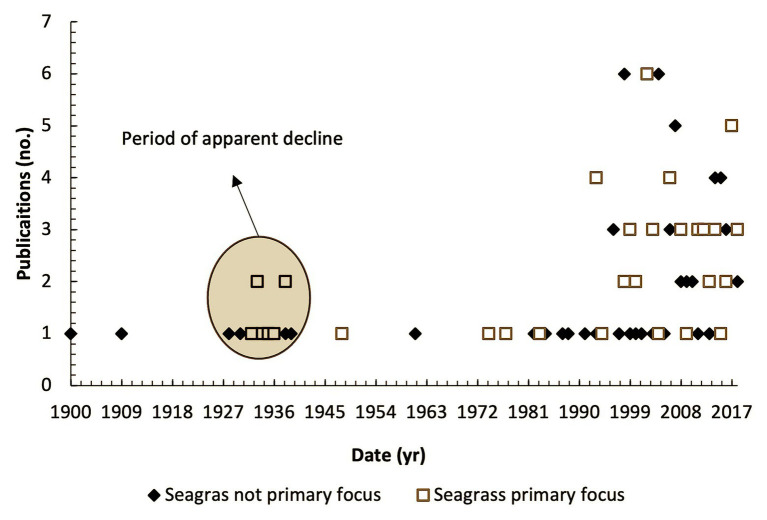
Number of publications (grey and peer reviewed) relating to United Kingdom seagrass habitats by date where seagrass is primary focus (orange square) and seagrass is significant secondary focus (blue diamond).

The first published account of seagrass in the United Kingdom that we are currently aware of was in 1831 (Winch, 1831), where it was included in a publication on the “*Flora of Northumberland and Durham*.” This observation is from a location on the Tyne River long since reclaimed and now an industrial estate.^1^ A peak in publications occurred around the time of the 1930’s wasting disease when naturalists became concerned with the substantial degradation of sites throughout the United Kingdom (Figure 3). Publications were sporadic until 1990 (*n* = 20) and since then have occurred more frequently as a series of peaks and troughs (*n* = 66).

Attempts to describe seagrasses across the United Kingdom were made by Butcher in 1932, but without any defined methodology (Butcher, 1934). He considered the occurrence of seagrasses regionally but did not provide area estimates. Seagrasses seemingly occurred ubiquitously, “apart from wave-swept, shingly and rocky shores to the west of the country” (Butcher, 1934). The abundance of seagrasses in sheltered and protected areas on the east coast was noted, as were plentiful populations in the lochs of Ireland and the west of Scotland (Butcher, 1934). Aside from this early attempt, efforts were made to document the status of seagrasses in Scotland in 1933 (Cleator, 1993), in Wales (Kay, 1998), and in Cornwall and the Isles of Scilly in 2002 (Hocking and Tompsett, 2002a,b). These reports provide presence and absence data and indicate widespread declines, but do not provide usable spatial estimates of area.

A full analysis and synthesis of the systematic search is provided in Supplementary Information. The literature analysed through the systematic review show ubiquitous declines across almost every region of the United Kingdom. Areas where good historic data are available show declines of between 40% (Cornwall) and 100% (Suffolk; Table 3). Although historic quantitative data are rare, the ubiquitous declines in seagrass areal extent are evident. Further, these historic declines are matched by pervasive recent declines, which suggest we are yet to stem this trend.

### Modelling the Loss of Seagrass Throughout England, Scotland, and Wales

The proportion of seagrass area per hectare of sub- and intertidal mud- /sandflat ranged from 4 to 86% with an average of 32 ± 27%. The 1991 NCC report (Davidson et al., 1991) established that estuaries comprised a total of 530,000 ha of coastal waters in mainland Britain. Of these, half are in England and almost one third are found within Scottish waters (Davidson et al., 1991). Within these, mud- /sandflats make up about 43%, totaling 254,400 ha (Davidson et al., 1991). The OSPAR dataset does not include any data from Ireland and is lacking in Scottish datapoints. It reports 143,571 ha of sub- and intertidal mud- /sandflats in mainland Britain, including those outside of estuaries. Considering one third of estuaries is found in Scotland it is unsurprising that these figures do not align. The total current mapped areal extent of seagrasses in mainland Britain (from this papers data) is 6,760 ha.

**Figure 4:**
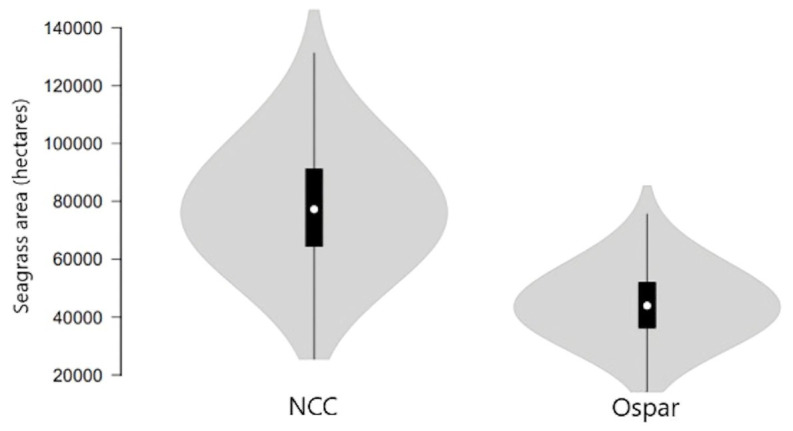
Modelled maximum seagrass area extent in mainland Britain from the NCC report (69) and the OSPAR dataset. Error bars indicate 95% CI.

Using the NCC data on total mud- /sandflats area, the estimated maximum seagrass extent for mainland Britain is 81,953 ha, with an upper 95% CI ranging from 126,430 to 40,964 ha (Table 5; Figure 4). Using the OSPAR data on total mud- /sandflats area, the maximum seagrass extent for mainland Britain is 43,559 ha, with 95% CI ranging between 72,647 and 24,267 ha (Table 5; Figure 4). Statistical comparisons between the two seagrass coverage values were made by comparing 95% CIs. Overlapping CIs indicated no significant difference between area estimates. The modelled data suggests that, with low certainty, between 36,799 and 75,193 ha of seagrasses has been lost from mainland Britain, this would represent an 84 and 92% decline, respectively (Table 3).

**Table 5:** Modelled maximum seagrass area extent, area and percentage loss in mainland Britain from the Nature Conservancy Council (NCC) report (Davidson et al., 1991) and the OSPAR dataset.

	NCC total mud- /sandflat area ha			OSPAR total mudflat area ha		
	254,400			143,571		
	Maximum seagrass extent ha	Seagrass loss ha	% decline	Maximum seagrass extent ha	Seagrass loss ha	% decline
Average	81,953	75,193	92	43,559	36,799	84
Upper 95%	126,430	119,670	95	72,647	65,887	91
Lower 5%	40,965	34,205	83	24,267	17,507	72

Average and 95% confidence interval displayed.

## Discussion

This study, to the best of our knowledge, is one of the first to systematically estimate the current and historic extent of seagrasses in any country and place it in the context of associated ecosystem services (see also, Ruiz et al., 2015; Harcourt et al., 2018). With high certainty, at least 44% of United Kingdom’s seagrasses has been lost since 1936. Of this, 39% has been lost since the 1980’s, which is substantially more than the suspected global decline of 29% in the same period (Waycott et al., 2009; Short et al., 2010). It also provides a different narrative to that emerging from the recent European review of seagrass status and trends that focused exclusively around monitoring data mostly from the previous 10 to 15 years (de los Santos et al., 2019). With medium certainty, 48% of seagrasses have been lost since 1936, 44% since the 1980s. The modelled potential distribution of seagrasses suggests with low certainty that up to 92% of seagrass has been lost from mainland United Kingdom waters.

We provide estimated loss ranges because the paucity of survey data means it is impossible to know exactly how much seagrass has been lost from these waters. Our high certainty estimates are almost certainly under-representative of the true scale of occurred losses. They only represent those meadows that have been documented, and almost all of these would have undergone some level of degradation prior to their documentation. Our model has limitations, but without data, it is an important step to understanding the wide-scale losses that have occurred. In consultation with ABPmer (Defra, 2020) the EA undertook a suitability model to assess where seagrasses could occur in United Kingdom waters. They documented 43,346 ha of suitable habitat in England alone. Based on the current areal extent of seagrasses in England (3,873 ha), this would represent a 91% loss. A similar suitability model was conducted for Wales, which indicated 4,541 ha of suitable habitat (Brown, 2015). Based on the current areal extent of seagrasses in Wales (551 ha), this would represent an 88% loss. Together, these models suggest a total of 47,888 ha of suitable seagrass habitat in England and Wales. This total is comparable to our lower estimate for the whole of mainland Britain (43,559 ha). Considering the suitability models and our lower estimate do not include any or many data points from Scotland, it would be reasonable to assume that the actual number is much closer to our higher estimate (81,953 ha). The EA also undertook a modelling project to map the historic areal extent and loss of saltmarsh habitats in England. Digitally overlaying ordinance survey maps from 1860, they combined these with historic maps of saltmarsh extent, and estimated coastline flooding capacity using LIDAR data to calculate an historic areal extent estimate of 215,624 ha (Mike Best EA, 2019, *personal communication*). This represents an 85% reduction on current saltmarsh extent in England. Although our modelled estimates may seem high, they are seemingly not out of character with other estimates of coastal degradation in the United Kingdom. Oyster reefs too are thought to have suffered similar extensive declines (Thurstan et al., 2013). If 85% of saltmarsh habitat has been lost in the United Kingdom, then the likelihood is that the environment which fringes it has also experienced widespread declines. The reef function of Oysters and their capacity to filter vast quantities of water rapidly means their loss would have had a significant negative impact upon the environmental conditions (light and shelter) of many areas that would have historically contained seagrass.

This study brings records together from disparate sources and provides the most up to date and accurate estimates of seagrass cover possible. The large-scale loss of seagrasses described here redefines the severity and spatial extent of what is known about biodiversity loss in our coastal seas, setting a new baseline upon which future management and restoration (Valdez et al., 2020) can aspire to build. Given the need to restore and improve management of these ecosystems, in light of work highlighting their importance to United Kingdom fisheries (Bertelli and Unsworth, 2014) and carbon sequestration (Green et al., 2018), and work highlighting the declining state of United Kingdom seagrass meadows (Jones and Unsworth, 2016), this work is much needed and timely in its arrival. Not all United Kingdom seagrass meadows has been lost and degraded, our research finds seagrasses persisting at many sites across the United Kingdom, to varying degrees of extent, with occurrences of seagrass recovery at some sites.

The rare accounts of documented areal extent of seagrasses in the early 1900’s provide an example of the changes that have likely occurred throughout the United Kingdom. Although these data are in isolation, the consistent declines noted in the literature, along with the documented 96% decline in average meadow size, confirms the trend of degradation pointed to by earlier studies (Butcher, 1934, 1941). Historic declines since the 1900’s are mirrored by more recent declines noted in the last decade (Jackson et al., 2016) and numerous incidences of small-scale disturbances in recent years (Unsworth et al., 2017).

There is strong evidence to suggest that seagrass loss can lead to a state of negative feedback preventing ecosystem recovery (Maxwell et al., 2017). However, this has not been the case for all United Kingdom sites. Intertidal (but not subtidal) recoveries observed in Milford Haven, Wales, where historic pollution encroachment and oil spills had previously reduced seagrasses (Bertelli et al., 2018), indicate removing or reducing stressors can, in some locations, lead to habitat recovery. The recovery of other intertidal seagrasses in the Leigh Marshes in the Greater Thames Estuary further supports this observation. History suggests meadows are capable of fluctuating and can recover from dramatic losses. The ability of seagrass meadows to regain abundance is encouraging and should help spur conservation initiatives globally, especially current attempts to promote seagrass restoration (Valdez et al., 2020).

### Understanding the Trends of Seagrass Decline in the United Kingdom

Rationalising the probable causes of such vast losses of seagrass in Britain is at best difficult, mostly because robust estimates regarding historic spatial extent of *Zostera* are limited. Typically, the seagrass wasting disease *Labyrinthula* has been described as the primary cause of virtually all seagrass loss in the United Kingdom (Butcher, 1934; Cottam, 1934; Den Hartog, 1983; Garrard and Beaumont, 2014). We argue that this assumption is itself a result of SBS. This assumption stems from the discussion within Butcher’s report, where undoubtedly seagrasses were lost due to disease (Butcher, 1934). Butcher reported changes to habitat based on his own intertidal experiences of seagrass abundance, supported by those of individuals whose baselines only go as far back as their own inherited knowledge (Butcher, 1934; Pauly, 1995). This case of SBS means the basis on which Butcher referred to healthy populations of seagrasses is likely a gross underrepresentation of what once occurred in these waters and is an excellent example of how SBS impacts contemporary environmental knowledge (Butcher, 1934). There has been no enquiry as to whether the seagrass habitats Butcher was assessing were already heavily degraded, with almost all the literature pointing to this period for the cause of seagrass degradation (Butcher, 1934). Considering the early industrialisation of the United Kingdom and a long history of mining activity, it is almost certain that the systems Butcher assessed in 1930’s would have already undergone dramatic declines. His assumptions were also largely based on visits to sites on the eastern shores of England. It is likely that persistent gradual declines had been occurring for centuries before Butchers report and these have continued to the present day (Jackson et al., 2016; Jones and Unsworth, 2016; Unsworth et al., 2017; Jones et al., 2018).

As the first country to industrialise in the 17 and 18th centuries, Britain had been undergoing dramatic land-use transformation long before Butcher assessed the status of seagrasses (Butcher, 1934). Industrialisation is intrinsically linked to environmental degradation. By the time, Butcher had been writing, dramatic physical alterations to the United Kingdom landscape had been occurring for at least 300 years (Butcher, 1934, 1941). A reference to seagrasses in the Tyne estuary in the early 1800’s (Winch, 1831) referred to a site that has since been reclaimed, now containing an industrial estate. Coastal reclamation, dredging and building of sea walls were prevalent in the 17th century, 200 years prior to this account (Batty, 1997), and are highly likely to have been as in conflict with seagrass as they still are today (Erftemeijer and Lewis, 2006). Importantly, the UK was at the forefront of the global metal industry, with metal mining prevalent throughout many parts of the United Kingdom during the 1700s and 1800s, with many of these mines (e.g., Wales) still producing extensive metal contaminated discharge into coastal and estuarine waters today. The negative impacts of a suite of heavy metals, causing toxic conditions for seagrasses, are well documented (Prange and Dennison, 2000; Macinnis-Ng and Ralph, 2002). Many areas thought to be viable seagrass sites within Welsh habitat suitability models are areas of historic heavy metal mining contamination, such as NE Anglesey (Whiteley and Pearce, 2003).

In addition to industrial development, the scale of overfishing of oysters around the United Kingdom cannot be ignored as a fundamental change and major disturbance to the environmental conditions. There is growing evidence of the close positive interactions that occur between seagrasses and many bivalves (Perkins, 1988; Gagnon et al., 2020). Locations such as the Firth of Forth have entirely lost up to 5,000 ha of oyster beds (Thurstan et al., 2013). These oyster beds would have fundamentally influenced the volume of suspended particles in the water column and hence the water clarity, creating conditions suitable for photosynthetic production by seagrasses. Similar estimates are available for areas, such as the Solent, the Thames, The Clyde, the Humber, and the Severn (Thurstan et al., 2013). The extraction activities of these Oyster fisheries were also likely to have disturbed and remobilised sediments at a high frequency over major areas for prolonged periods, potentially negatively impacting seagrasses. It is not only Oyster fisheries that would have likely had a historic negative impact upon seagrass. As early as the 1700s, bottom trawling was already widespread around the coastal waters of the Great Britain (Jones, 2018) and such activity is well known to directly damage seagrasses (Blaber et al., 2000). Seagrasses remain threatened in the United Kingdom today by activities which reduce water quality, and direct physical disturbance (Jones and Unsworth, 2016; Green et al., 2018).

### The Need for Improved Seagrass Assessment

The present analysis highlights a lack of coherent and systematic monitoring and mapping programmes of seagrass meadows in the United Kingdom. That 64% of records in the OSPAR dataset are older than 20 years highlights the prolific lack of constant effort in seagrass mapping. Despite this fact, the OSPAR dataset is the baseline for estimates of United Kingdom seagrass extent included by the UNEP World Conservation Monitoring Centre (WCMC). Since this is the first attempt to provide an accurate map and up-to-date map of seagrass occurrence and declines for an entire country it is likely that these are not the only data included by WCMC that are inaccurate (McKenzie et al., 2020). The paucity of current data means that estimates, even coarse ones, are a necessary step to evaluating the pressures imposed on seagrasses, in keeping with the accepted approach of “*use available data*” (Hiscock, 1997). However, data inconsistencies can make it challenging to talk meaningfully about global seagrass trends and arguably managers should only be working with temporal and spatial data that we are reasonably confident is accurate. Regional and local mapping of sites around the world is important in ensuring that current attempts to increase seagrass abundance through restoration, rehabilitation, and conservation have any hope to succeed.

**Figure 5:**
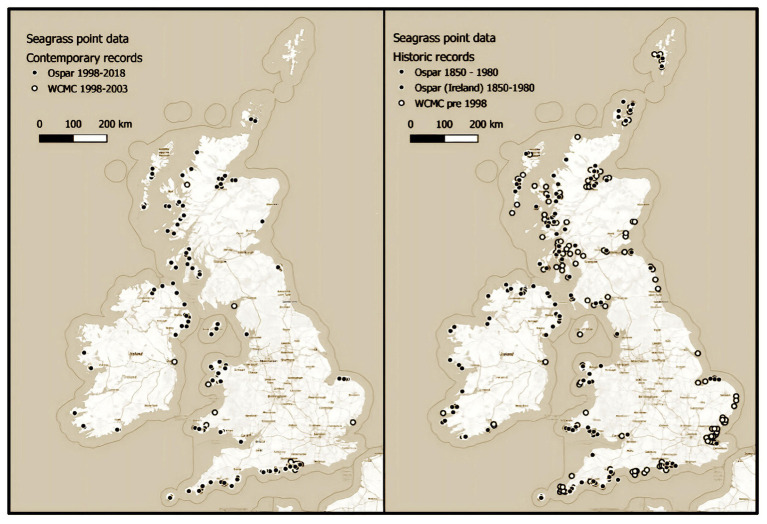
Seagrass point data from the OSPAR and UNEP-World Conservation Monitoring Centre datasets showing pre-1998 surveys (left) and post-1998 surveys (right).

Data inconsistencies found within this work are most obvious in Scotland, where most data were collected over 20 years ago. Although the isolation from human population likely means there is potential for these meadows to remain intact, many have likely been impacted by the extensive and continually expanding salmon aquaculture industry, with fish farming a known cause of seagrass loss in other parts of the world (Berry and Davison, 2001). The meadows where historic and contemporary data are available show mass declines. The once huge meadows in the Cromarty and Dornoch Firths have been reduced from 3,241 to 1,200 ha and 2,546 to 117 ha, respectively. Regardless, huge swathes of Scottish waters have not been surveyed for over 2 decades and could represent a vital stronghold of this once ubiquitous United Kingdom habitat. Their condition and extent should be assessed with urgency. Scotland is not the only region where survey efforts since 1998 have been insubstantial. Pre- and post-1998 maps show a reduced survey effort across all regions (Figure 5).

### Impact of Declines on the Ecosystem Services Afforded by United Kingdom’s Seagrass

Recent efforts have been made to estimate the amount of carbon stored within the United Kingdom seagrass meadows (Röhr et al., 2018; Green et al., 2018; Lima et al., 2020). These papers analysed sediment from 13 meadows along the southwest coast of the United Kingdom for organic carbon content. For areas around the Solent and adjacent harbours, Lima et al. (2020) found 33.8 ± 18.5 Mg C ha^−1^ in the top 30 cm of sediment. Whilst Green et al. (2018) found meadows contained a total of 141 ± 73 Mg C ha^−1^, within the top 1 m of seagrass sediment. We have used mean values across both United Kingdom seagrass species as current carbon values are limited and explicit assessment of the impact of species and environmental variation upon this are largely absent. Based on these average figures, the estimated total carbon stored in the top 100 cm of recently mapped seagrasses of the United Kingdom is ~1.2 Mt carbon. In mainland Britain, this figure is 0.9 Mt of carbon (Table 5). Based on the upper (81,952 ha) and lower (40,965 ha) estimates of historic seagrass distribution, mainland United Kingdom seagrass meadows could once have contained between 5.7 and 11.5 Mt of carbon (Table 5). The upper value of this is equivalent to 3% of the United Kingdoms CO_2_ emissions in 2017 (Eaton, 2019).

There exists only one value currently of United Kingdom seagrass carbon sequestration, but this value itself is an estimate (Garrard and Beaumont, 2014), hence there are no reliable estimates of seagrass sequestration rates in the United Kingdom. Reasonable and frequently used rates in the literature give low (0.044 cm yr.^−1^), medium (0.202 cm yr.^−1^), and high (0.42 cm yr.^−1^) bounds to frame carbon sequestration estimates (Duarte et al., 2013; Lavery et al., 2013; Macreadie et al., 2013; Miyajima et al., 2015; Röhr et al., 2018). Here, sequestration rates were estimated by dividing the total carbon estimates by the amount of time it takes to accumulate this stock using the sedimentation rates above, to provide estimates on average annual carbon accumulation of United Kingdom seagrass meadows (Lavery et al., 2013; Röhr et al., 2018; Table 6.). Assuming a medium sedimentation rate, the seagrass meadows of the United Kingdom are accumulating roughly 0.024 Mt C yr^−1^ (Table 6). Assuming this medium sedimentation rate, historic undisturbed seagrass meadows of the United Kingdom could have been absorbing 0.232 Mt C yr^−1^ (Table 6).

**Table 6:** Estimates of total carbon (Mt C) of modeled historic and contemporary seagrass distribution of mainland Britain, and of contemporary seagrass distribution of the United Kingdom, with low (0.044 cm yr^−1^), medium (0.202 cm yr^−1^), and high (0.42 cm yr^−1^) estimates for carbon sedimentation per year (Mg C yr.^−1^).

	Carbon stock		Sedimentation rates		
	Area ha	Total carbon Mt	Low Mt. C yr^−1^	Medium Mg C yr^−1^	High Mg C yr^−1^
Upper historic estimate	81,953	11.5	0.050	0.232	0.483
Lower historic estimate	40,965	5.7	0.025	0.115	0.239
Contemporary area United Kingdom	8,493	1.2	0.005	0.024	0.050
Contemporary area mainland Britain	6,760	0.9	0.004	0.018	0.038

Considering the need to include natural ecosystems in climate mitigation strategies, there is increasing interest in placing monetary valuations on carbon stock and sequestration estimates. The United Kingdom government has recently implemented a legal commitment to achieve Net-Zero greenhouse gas emissions by the year 2050. To reach this target will require major economic reforms and substantial increases in natural carbon sequestration capacity. The current United Kingdom carbon value is £24/t C (DECC, 2011; Green et al., 2018). However, according to the Grantham Institute, a price that is consistent with the Net-Zero targets needs to begin at £50/t C, rise to £75/t C in 2030, and to £160/t C in 2050 (Burke et al., 2019). Based on today’s market price, the value of the carbon stored in the top 100 cm of recently mapped seagrass stands at a value of £29 million with yearly (medium) sequestration value (Table 6) of £0.58 million, rising to £3.9 million over the next 30 years, if the projections hold true (Table 7). Taking the upper (Table 6) range of the historic estimates of seagrasses in mainland Britain, at today’s market value, these would once have contained £276 million worth of carbon in their sediments. In this undisturbed state, these seagrass meadows could have been responsible for sequestering £5.6 million worth of carbon every year, rising, if the projections hold true, to £37.2 million over the next 30 years (Table 7). These figures, although crude, offer a powerful indicative snapshot of what has been lost through long-term environmental degradation, and support the need to offer protection to those seagrass meadows that remain. Seagrasses use to be ubiquitous along the shores of the United Kingdom, and if restored to even part its former extent; this ecosystem will provide valuable support to reaching a carbon neutral future.

**Table 7:** Estimates of total carbon (Mt C) and current and projected increases in carbon economic value (£million) of modelled historic and contemporary seagrass distribution of mainland Britain, and of contemporary seagrass distribution of the United Kingdom, medium (0.202 cm yr^−1^) estimates for carbon sedimentation per year (Mg C yr.^−1^), and associated current and projected increases in carbon economic value (£million).

	Total market value £million					Total market value £million			
	Area	Total carbon	Today	NZR Today	30 years	Sequest-ration	Today	NZR Today	NZR 30 years.
	*ha*	*Mt*	£24/t C	£50/t C	£160/t C	*Mg C yr^−1^*	£24/t C	£50/t C	£160/t C
Upper historic estimate	81,953	11.5	276	575	1840	0.23	5.6	11.6	37.2
Lower historic estimate	40,965	5.7	136.8	285	912	0.12	2.8	5.8	18.4
Contemporary area United Kingdom	8,493	1.2	28.8	60	192	0.02	0.6	1.2	3.9
Contemporary area mainland Britain	6,760	0.9	21.6	45	144	0.02	0.4	0.9	2.9

NZR, net zero requirement.

The value that seagrasses provide for other ecosystem services cannot be readily quantified financially with current data; however, the functions they play are extensive in the United Kingdom, particularly with respect to fisheries support, biodiversity, nutrient cycling, and sediment stabilisation (Nordlund et al., 2016). United Kingdom studies have revealed that seagrass harbours 4.6 times the abundance of fish of unvegetated habitat at a density of 6,000 fish per hectare (Bertelli and Unsworth, 2014), resulting in United Kingdom seagrasses currently supporting approximately 50 million fish, many of commercial importance (e.g., juvenile whiting, cod, plaice, and pollack). Based on the upper (81,952 ha) and lower (40,965 ha) estimates of historic seagrass distribution, mainland United Kingdom seagrass meadows could once have contained between 200 and 400 million fish, a potential loss of 274 million fish based on the comparison to unvegetated seabed.

Given the eutrophication issues faced by many coastal waters around the United Kingdom, the loss of seagrasses will have had a detrimental impact upon water quality. For example, *Z. marina* meadows may cycle approximately 49 kg of nitrogen per hectare year (Watson et al., 2020). This equates to the current annual cycling of 416 tonnes per year that could have historically been as high as 4,015 tonnes per year. Seagrasses also help reduce coastal erosion through improved stability of sediments. The extent of this role is often context dependent (Ondiviela et al., 2014) but available evidence indicates that at high density *Z. marina* may increase by up to 10 fold the sheer strength of the sediment (Widdows et al., 2008). Given the vast loss of seagrasses in eastern parts of the United Kingdom in areas know to be subject to coastal erosion and predicted to suffer from increasing impacts of rising sea levels, we hypothesise that at its historic extent seagrasses would have played a pivotal role in reducing coastal erosion.

## Conclusion

Although the United Kingdom has arguably been altering its natural habitats for longer than almost any other country, the trends and impacts of declines exposed in this paper are likely occurring in many other developing and developed counties. This analysis shows the devastating ecosystem services losses that this decline has caused. It is hoped that this paper will not only generate a better understanding of seagrass losses in the United Kingdom, but also spur efforts to protect remaining seagrasses and restore historical losses and drive other countries to take stock of this vital coastal habitat to the same goal.

## Data Availability Statement

The seagrass distribution data used in this study have been deposited in the Dryad Digital Repository (Green et al., 2021).

## Author Contributions

All authors conceived the ideas and designed the methodology. AG collected the data and led the writing of the manuscript. AG and MC analysed the data. All authors contributed critically to the drafts and gave final approval for publication. All authors are accountable for all aspects of the work in ensuring that questions related to the accuracy or integrity of any part of the work are appropriately investigated and resolved. All authors contributed to the article and approved the submitted version.

### Conflict of Interest

The authors declare that the research was conducted in the absence of any commercial or financial relationships that could be construed as a potential conflict of interest.

## Footnotes

^1^https://seagrassspotter.org/sighting/1816
